# Body Mass Index Thresholds and the Use of Bariatric Surgery in the Field of Kidney Transplantation in Germany

**DOI:** 10.1007/s11695-022-06000-4

**Published:** 2022-03-19

**Authors:** Tomasz Dziodzio, Karl Herbert Hillebrandt, Sebastian Knitter, Maximilian Nösser, Brigitta Globke, Paul Viktor Ritschl, Matthias Biebl, Christian Denecke, Jonas Raakow, Georg Lurje, Wenzel Schöning, Moritz Schmelzle, Andreas Kahl, Markus Fütterer, Klemens Budde, Kai-Uwe Eckardt, Fabian Halleck, Johann Pratschke, Robert Öllinger, German Bariatric Surgery, Kidney Transplantation Group, Assfalg Volker, Bachmann Anette, Benoehr Peter, Billeter Adrian, Braun Felix, Brockmann Jens, Er Aydin, Foller Susan, Friedersdorff Frank, Fuehrer Andreas, Geks Josef, Grabitz Klaus, Guthoff Martina, Gwinner Wilfried, Halleck Fabian, Heise Michael, Herden Uta, Karakizlis Histros, Keese Michael, Kersting Stephan, Koch Martina, Krautter Markus, Kuhlmann Uwe, Kurschat Christine, Lang Sven, Liefeldt Lutz, Lokhande Shanti, Lopau Kai, Manekeller Steffen, Mönch Christian, Mühlfeld Anja, Nadalin Silvio, Nitschke Martin, Öllinger Robert, Opgenoorth Mirian, Pein Ulrich, Pession Ursula, Pisarski Przemyslaw, Putz Juliane, Rath Thomas, Schenker Peter, Seehofer Daniel, Siemer Stefan, Sommer Florian, Stroehlein Michael, Treckmann Jürgen Walter, Weinmann-Menke Julia, Weithofer Peter, Wiegand Karl, Zecher Daniel

**Affiliations:** 1 Department of Surgery, Campus Charité Mitte - Campus Virchow-Klinikum, Charité - Universitätsmedizin Berlin, Augustenburger Platz 1, 13352 Berlin, Germany; 2 BIH Charité Clinician Scientist Program, Berlin Institute of Health (BIH), Berlin, Germany; 3 Department of Nephrology and Medical Intensive Care, Charité Universitätsmedizin Berlin, Berlin, Germany; 4 Department of Endocrinology and Metabolic Diseases, Charité Universitätsmedizin Berlin, Berlin, Germany

**Keywords:** Obesity, End-stage renal disease, Kidney transplantation, Bariatric surgery

## Abstract

**Background:**

Obesity in the recipient is linked to inferior transplant outcome. Consequently, access to kidney transplantation (KT) is often restricted by body mass index (BMI) thresholds. Bariatric surgery (BS) has been established as a superior treatment for obesity compared to conservative measures, but it is unclear whether it is beneficial for patients on the waiting list.

**Methods:**

A national survey consisting of 16 questions was sent to all heads of German KT centers. Current situation of KT candidates with obesity and the status of BS were queried.

**Results:**

Center response rate was 100%. Obesity in KT candidates was considered an important issue (96.1%; *n* = 49/51) and 68.6% (*n* = 35/51) of departments responded to use absolute BMI thresholds for KT waiting list access with ≥ 35 kg/m^2^ (45.1%; *n* = 23/51) as the most common threshold. BS was considered an appropriate weight loss therapy (92.2%; *n* = 47/51), in particular before KT (88.2%; *n* = 45/51). Sleeve gastrectomy was the most favored procedure (77.1%; *n* = 37/51). Twenty-one (41.2%) departments responded to evaluate KT candidates with obesity by default but only 11 (21.6%) had experience with ≥ *n* = 5 transplants after BS. Concerns against BS were malabsorption of immunosuppressive therapy (39.2%; *n* = 20/51), perioperative morbidity (17.6%; *n* = 9/51), and malnutrition (13.7%; *n* = 7/51).

**Conclusions:**

Obesity is potentially limiting access for KT. Despite commonly used BMI limits, only few German centers consider BS for obesity treatment in KT candidates by default. A national multicenter study is desired by nearly all heads of German transplant centers to prospectively assess the potentials, risks, and safety of BS in KT waitlisted patients.

**Graphical abstract:**

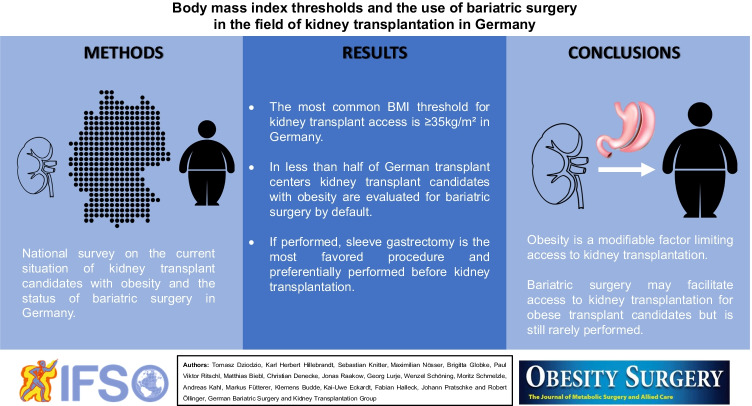

**Supplementary Information:**

The online version contains supplementary material available at 10.1007/s11695-022-06000-4.

## Introduction

The global pandemic of morbid obesity is strongly associated with the metabolic syndrome and related comorbidities like hypertension, atherosclerosis, and type 2 diabetes mellitus and affects 23% of the German population [1]. Each of abovementioned diagnoses alone or in combination is linked to increased morbidity and inferior patient survival and can aggravate chronic kidney disease (CKD) and the progression to end-stage renal disease (ESRD) [2–4]. Kidney transplantation (KT) is the only curative treatment of ESRD [5]. In KT, obesity is associated with higher rates of delayed organ function (DGF), primary non-function (PNF), wound complications, and poor graft survival [6, 7]. Therefore, most international and national transplant societies recommend weight reduction in KT candidates with morbid obesity to improve transplant outcome [8, 9]. However, sustainable conservative weight loss is hard to achieve and most KT candidates rather gain weight on chronic dialysis [10, 11]. The persistent organ shortage and the pressure to fulfill transplant quality metrics force many transplant centers to limit access to transplant waiting lists by utilizing institutional maximum body mass index (BMI) thresholds [12]. Bariatric surgery (BS) has been established as a superior treatment option for morbid obesity in the non-ESRD population and may also present a potential therapy for KT candidates with obesity [6, 13]. Although several centers offer BS in KT candidates, it is still not considered a standard treatment option in these patients. The aim of our study was to establish an overview of the current practice patterns of obesity management and the role of BS in the field of KT in Germany.

## Methods

### Data Acquisition

A web-based survey consisting of 16 questions was designed (Google Forms, Google Inc.) and submitted to all 39 German kidney transplant centers. Both surgical and nephrological heads of transplant programs at each center were addressed. The questionnaire covered the following topics: the role and the current situation of obesity in KT candidates at the addressed centers, the use and range of weight-related waiting list/transplant access restrictions (BMI thresholds), the applied weight reduction programs and bariatric techniques, and concerns on and expectations related to BS in the field of KT.

### Statistical Analysis

All calculations and statistical analyses were performed using SPSS software package, version 25 (IBM, Armonk, NY, USA). Graphs were created using GraphPad Prism software version 8 (GraphPad Software, San Diego, CA, USA). Comparison of answers to the query between surgeons and nephrologists was performed using Pearson’s chi-square or Fisher’s exact test, as applicable. *P* values < 0.05 were considered statistically significant. The primary aim of the study was to provide a concise overview of the current situation of obesity and BS in KT candidates in Germany. The study does not compare or rate strategies, nor does it draw causal conclusions.

## Results

**Fig. 1:**
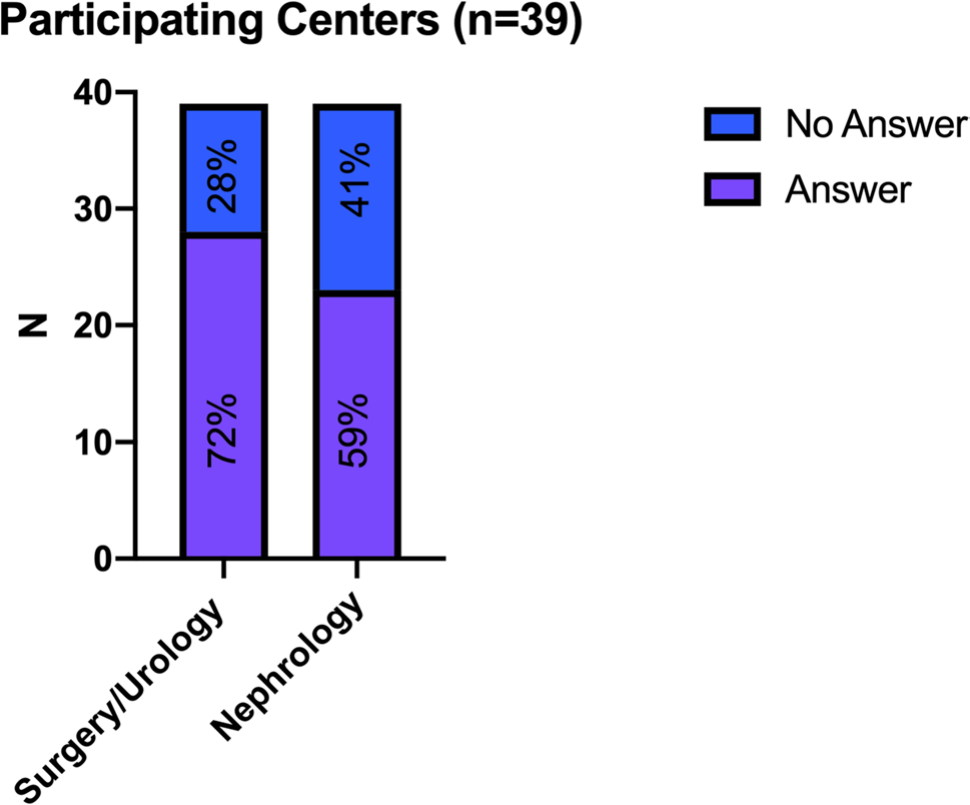
Center response rate shown as total and divided into nephrology and surgery

From all 39 German transplant centers, at least one responsible person responded to the questionnaire (response rate: 100%). Detailed results of the survey are shown in supplementary file 1. In 27 centers (69.2%), the reply to the query was performed by either the head of the nephrological (*n* = 11; 28.2%) or the surgical department (*n* = 16; 41%). In 12 centers (30.7%), heads of both departments responded. The response rate and distribution are shown in Fig. 1.

**Fig. 2:**
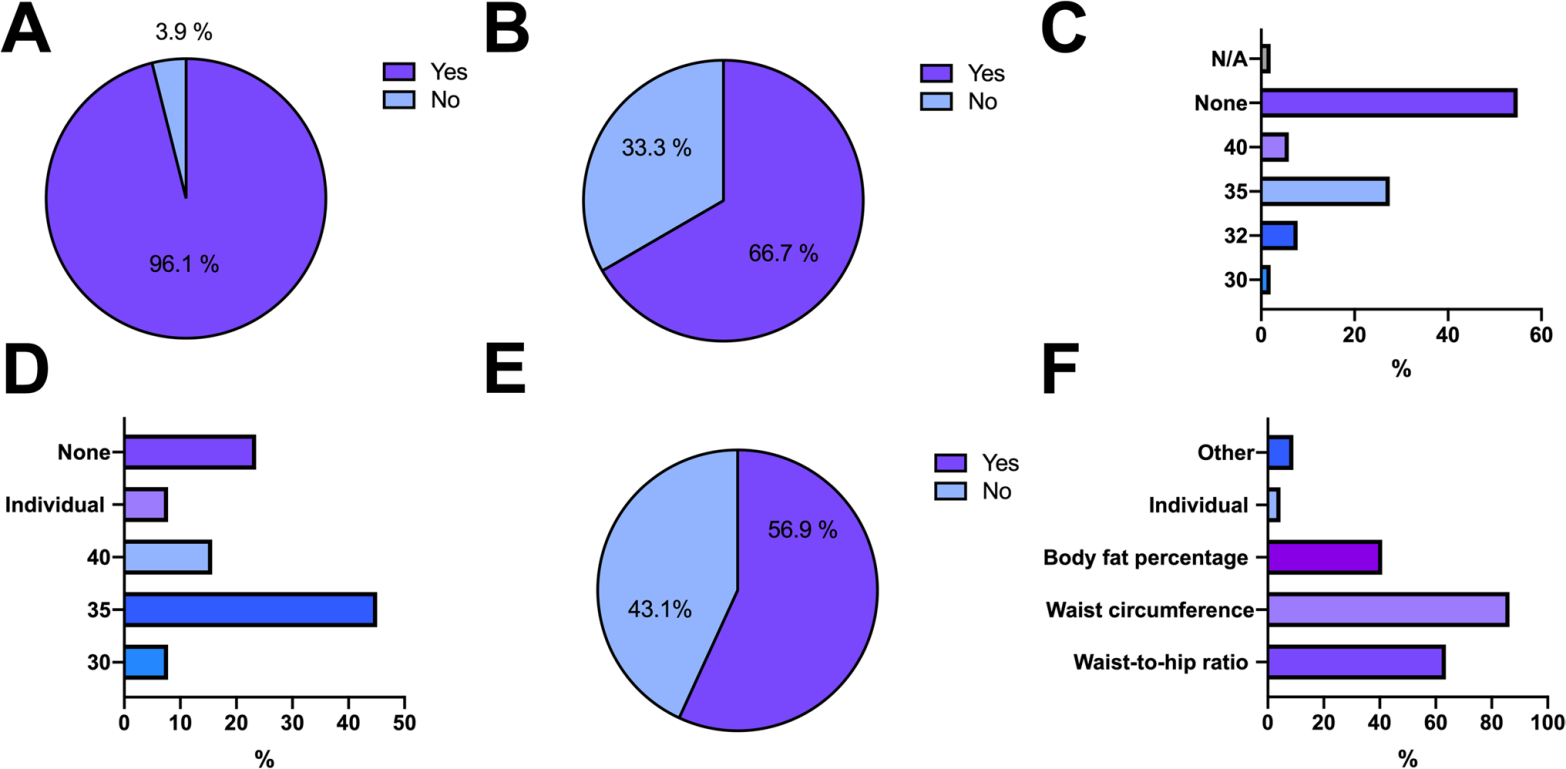
Survey questions and responses shown as total numbers or % (**A** Does obesity in ESRD patients present a relevant issue in your center? **B** Does your center have a policy or standard operating procedure for ESRD patients with obesity regarding inclusion on the kidney transplant waiting list? **C** What is the upper limit BMI at your center for considering patients with ESRD for kidney transplantation evaluation? **D** What BMI is the upper sole exclusion criterion for patients with ESRD to be eligible for kidney transplantation [status “transplantable”] at your center? **E** Does the BMI present an appropriate criterion for the selection of potential kidney transplant candidates? **F** If not, which of the following parameters do you consider as better criteria? [Multiple answers possible])

Obesity in KT candidates was considered to be a relevant issue in 96.1% (*n* = 49/51) of transplant departments and 66.7% (*n* = 34/51) stated to use obesity as a selection criterion for KT. In 27.5% (*n* = 34/51) of centers, a BMI ≥ 35 kg/m^2^ was considered a limit for KT evaluation, whereas 54.9% (*n* = 28/51) of centers did not consider obesity a contraindication evaluation for KT. Whilst 43.1% (*n* = 22/51) of transplant departments stated that the BMI is not an appropriate criterion for the selection of potential KT candidates, 68.6% (*n* = 35/51) reported to use BMI thresholds as sole exclusion criterion for KT listing. The most common BMI threshold for active KT listing was ≥ 35 kg/m^2^ in 45.1% of centers (*n* = 23/51). Only 8 (15.7%) centers considered active KT listing in candidates with a BMI ≥ 40 kg/m^2^. Nearly half of the centers (43.1%; *n* = 22/51) specified waist circumference (37.2%; *n* = 19/51), waist-to-hip ratio (27.4%; *n* = 14/51), and body fat percentage (17.6%; *n* = 9/51) as more appropriate alternative selection criteria for potential KT recipients compared to the BMI (multiple answers were possible; Fig. 2).

**Fig. 3:**
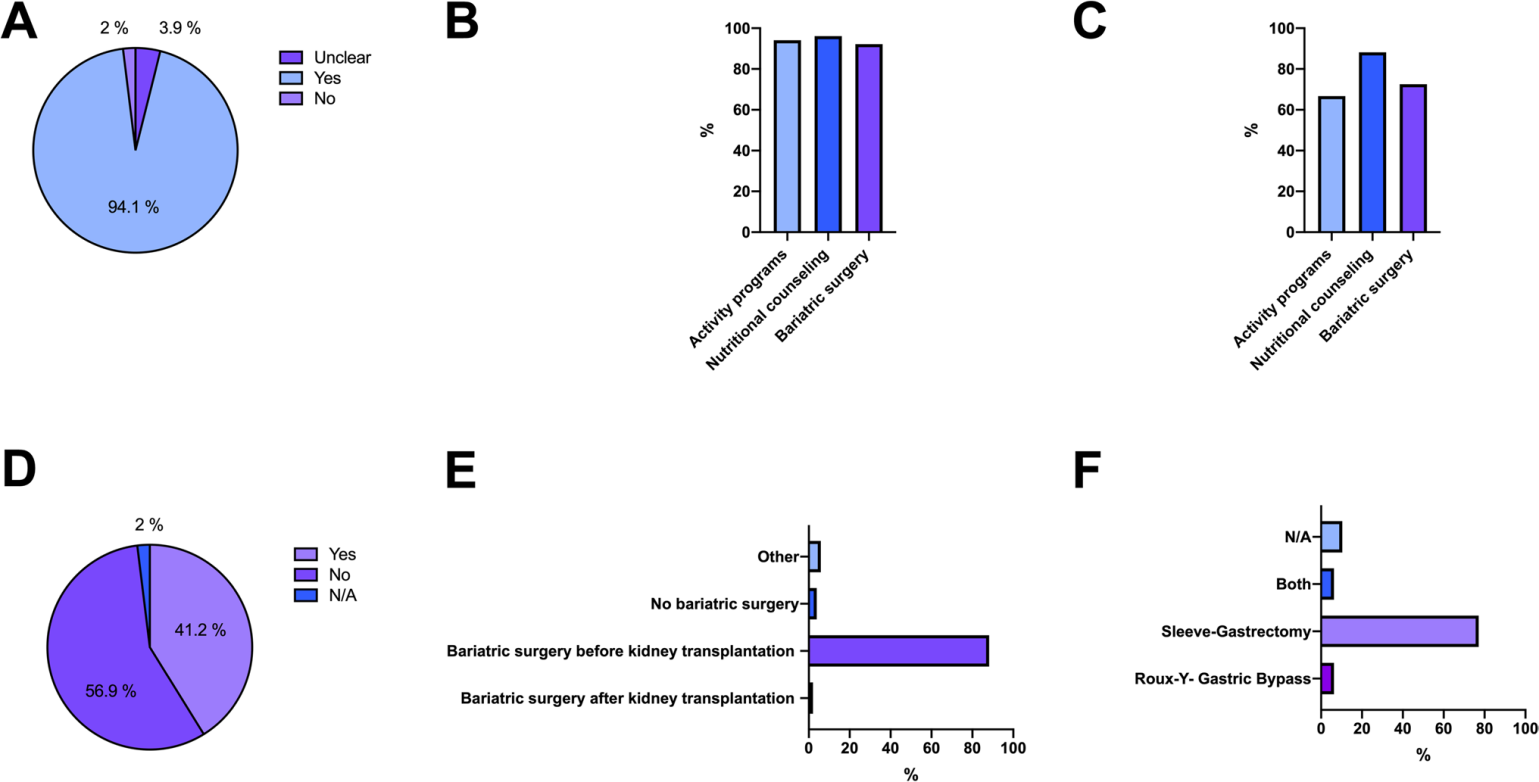
Survey questions and responses shown as total numbers or % (**A** Are weight loss programs useful measures for patients with obesity before kidney transplantation? **B** Which weight reduction measures do you consider appropriate in ESRD patients to treat obesity prior to kidney transplantation? [Multiple answers possible] **C** Which weight loss measures does your center offer to ESRD patients with obesity in preparation for kidney transplantation? [Multiple answers possible] **D** Do you treat all waiting list patients, who are above the BMI threshold with bariatric surgery at your center? **E** Which bariatric surgery is the most suitable in the context of kidney transplantation?)

**Fig. 4:**
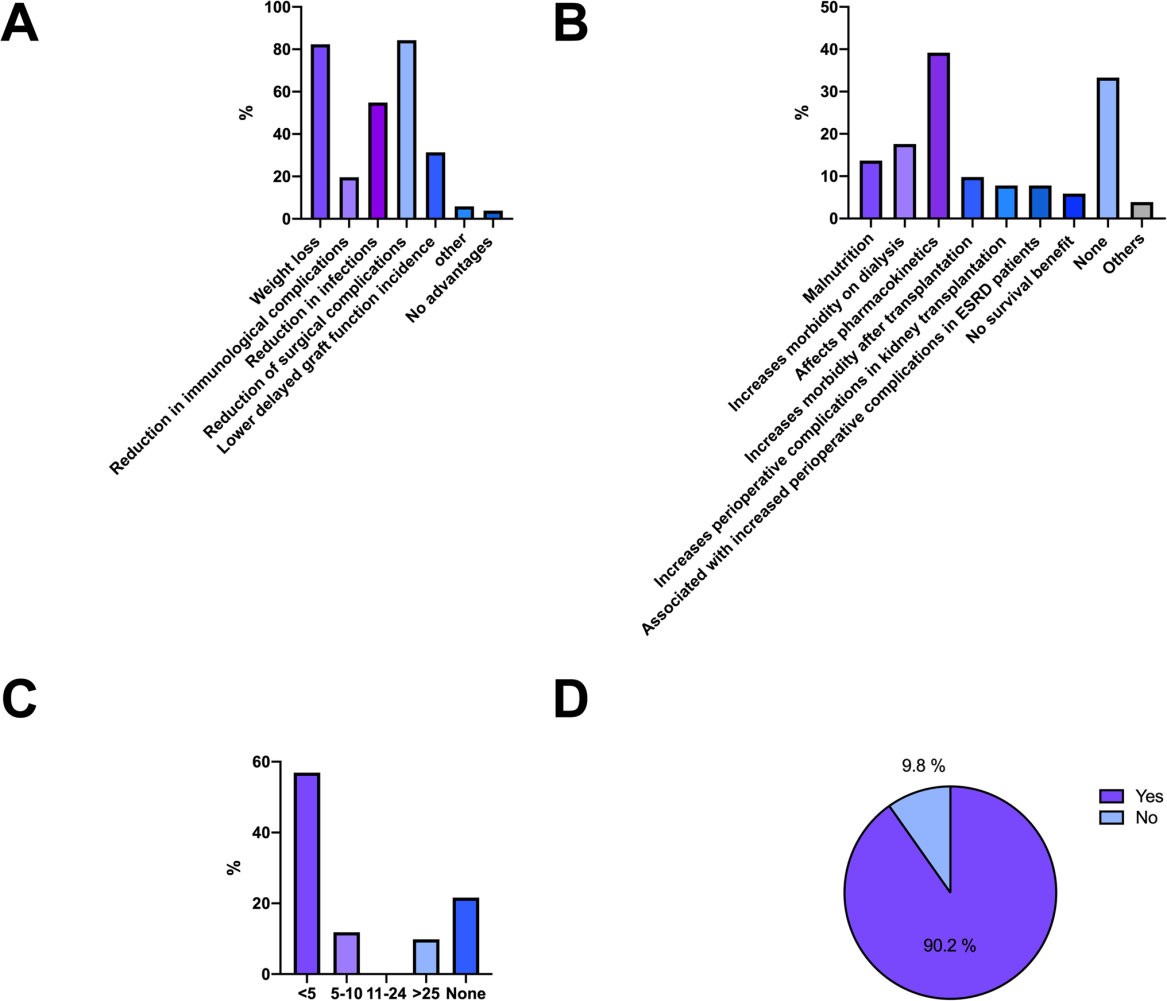
Survey questions and responses shown as total numbers or % (**A** What may be the benefits of bariatric surgery before kidney transplant? [Multiple answers possible] **B** What concerns do you have about bariatric surgery in the context of kidney transplantation [Multiple answers possible] **C** How many patients underwent kidney transplantation after bariatric surgery at your center? **D** Are you interested in participating in a prospective multicenter study on obesity in the context of kidney transplantation?)

Almost all centers (94.1%; *n* = 48/51) deemed interdisciplinary weight loss programs as useful to treat obesity in KT candidates: Nutritional counseling (96.1%; *n* = 49/51), activity programs (94.1%; *n* = 48/51), and BS (92.2%; *n* = 47/51) were all considered appropriate weight reduction measures. The actually offered weight loss programs included nutritional counseling in 88.2% (*n* = 45/51), activity programs in 66.7% (*n* = 34/51), and BS in 72.5% (*n* = 37/51) of centers. Most transplant centers (88.2%; *n* = 45/51) favored BS before KT, and in 41.2% (*n* = 21/51) of centers, BS was offered regularly to KT waiting list candidates exceeding the BMI threshold. Sleeve gastrectomy was considered the most favored technique (77.1%; *n* = 37/51; Fig. 3). The most frequently mentioned benefits of BS before KT were weight loss (82.4%; *n* = 42/51) and the reduction of surgical complications (84.3%; *n* = 43/51), infections (54.9%; *n* = 28/51), and DGF rates (31.4%; *n* = 16/51). Only 2 (3.9%) center heads expressed the opinion that BS before KT does not provide any benefits. The biggest concerns against BS before or after KT were potential pharmacokinetic alterations in immunosuppressive therapy (39.2%; *n* = 20/51), increased morbidity on dialysis (17.6%; *n* = 9/51), and malnutrition (13.7%; *n* = 7/51). Surgeons had not significantly less concerns with regard to BS for patients with obesity waitlisted for KT (surgery 42.9% [*n* = 12/28] vs. 21.7% [*n* = 5/23] nephrology, *p* = 0.196). Regarding the experience with BS in the context of KT, only 5 (9.8%) of transplant centers had performed more than 25 KTs after BS, 6 (11.8%) centers had KT experience with 5–10 KTs after BS, and 11 (21.6%) centers reported not yet having performed a KT after BS. The interest in participating in a prospective multicenter trial evaluating the effects of BS in KT and to develop national standards was expressed in 90.2% (*n* = 46/51) of centers (Fig. 4).

## Discussion

The growing discrepancy between organ demand and supply, an ageing population, the increase in marginal donors and recipients, and national or self-imposed quality standards are shaping the everyday life of transplantation medicine nowadays [12, 14, 15]. In 2020 a total of 7388 patients were listed for KT in Germany [16]. Obesity and especially morbid obesity have been reported to be associated with complications and impaired outcome in KT [17–19]. Hence, access to KT waiting lists is often restricted for high-risk ESRD candidates with obesity by BMI thresholds ranging from 35 to 45 kg/m^2^ [6, 12]. In 2012, Lentine et al. analyzed available recommendations and studies on obesity in the setting of KT and concluded that obesity in KT recipient is associated with inferior transplant outcome compared to KT recipients with normal body composition [8]. However, no data was found to support specific BMI thresholds and the authors concluded that the decision for each patient should be made individually depending on the overall condition of the patient. Additionally, most of the data used to argue waiting list access restrictions for KT candidates with obesity is based on studies from the 1990s and early 2000s [17, 20–22]. Several more recent studies have questioned the use of BMI thresholds [23, 24]. A literature review by Minh-Ha Tran et al. from 2016 found that albeit increased risk for surgical site infections and DGF rates in KT candidates with obesity compared to non-obese KT recipients, there was no impact on graft and patient survival [24].

Our survey found that obesity represents a relevant issue in nearly all German transplant centers (96.1%). Almost half of the centers considered BMI as an inappropriate criterion for KT selection (43%) and suggested to use waist-to-hip ratio or waist circumference as more proper parameters for obesity-related risk stratification. This assessment is supported by the results of a meta-analysis by Czernichow et al. [25]. The authors investigated the evidence of different obesity discriminators on cardiovascular disease mortality in 82,864 British citizens and found that measures of central adiposity (waist-to-hip ratio and waist circumference) were stronger predictors of cardiovascular mortality than BMI. At present, the reality looks different, and most centers worldwide still use BMI as a discriminator for obesity-related complications. This is most likely due to the fact that BMI measuring/updating is an easier task compared to the other discriminators. In our survey, more than half of German transplant centers (68.6%) reported to use BMI limits for KT access, albeit the rate was lower than in similar international questionnaires. In a survey of 67 KT centers in the USA, nearly all centers (99%) stated to use BMI thresholds for KT access, although the exact numbers and BMI limits have never been made publicly available [26]. A more recent query from Canada reported that 81% of transplant specialists use official obesity-related selection criteria for KT [27]. The authors found that if there was a BMI threshold, the most commonly used BMI limit for KT listing was 40 kg/m^2^ (62%), followed by ≥ 35 kg/m^2^ (36%). In our query, more than half of the centers had no BMI limit for waitlist evaluation. However, after waitlisting, most centers used BMI upper limits as a selection criterion for effective transplantation and the most common BMI threshold was ≥ 35 kg/m^2^. This result implies that there is a considerable number of patients on German KT waiting lists, who are denied KT after evaluation solely based on the diagnosis of morbid obesity. Interestingly, we found a consensus between surgeons and nephrologists concerning BMI limits for the access to the waiting list and the effective transplantation, although one would expect surgeons to be more restrictive.

Nearly all centers agreed that weight loss programs are useful measures for patients with obesity before KT. In our survey, 3 possible treatment approaches for weight reduction were eligible and we allowed multiple answering. There was unanimous consent that there is no single treatment option, but that a multimodal approach of nutritional counseling, activity programs, and BS seems appropriate. A discrepancy was found between the recommendation and what was actually offered to patients with obesity, especially for activity programs and BS. However, the availability of treatment options in German centers was considerably higher than that in the Canadian study, in which only 30% reported offering a weight loss program for KT candidates with obesity [27]. Nonetheless, our results indicate the potential to provide more personalized obesity treatment options to these patients.

In general, the reported BS expertise in KT candidates among German transplant centers was relatively low. The majority of centers had no BS experience or with less than 5 patients (78.5%). Only the minority oversaw more than 25 patients with BS prior to KT (10%). Interestingly, nephrologists (95.7%) were more open to considered BS an appropriate treatment option for ESRD patients with obesity than surgeons (89.3%); however, they had more concerns about BS than surgeons. Albeit nearly all centers agreed that BS should be offered before KT, less than half of them considered BS for obesity treatment in KT candidates by default. Based on the data of the current query, it is not possible to explain the actual causes of this discrepancy. The most common concern for BS in KT candidates were potential negative effects on pharmacokinetics of immunosuppressive medication and malabsorption, whereas increased perioperative complications after BS were rarely considered a relevant issue (7.8%). In 2017, we investigated the impact of BS on alterations of immunosuppressive therapy in a systematic literature review and found that most authors reported no or negligible effects of immunosuppressive therapy regimes [6]. Sleeve gastrectomy was considered the most favored procedure in our survey (77.1%) and Roux-Y-gastric bypass did not appear to play a relevant role in this patient population. This is probably motivated by the fact that sleeve gastrectomy is considered the safer procedure in this patient cohort with regard to general and surgical site complication with less malnutritional and malabsorptive effects on vitamins and minerals [28–30].

The use of bariatric surgery to treat obesity in patients with ESRD inhears the potential to enable a broader access to KT waiting lists by overcoming national or self-imposed BMI thresholds. In summary, this survey addresses many relevant issues regarding obesity and its treatment in the field of KT. On the one hand, we obtained the perspectives from many heads of nephrological and surgical transplantation programs with a center response rate of 100%. On the other hand, we observed that the experience with BS in KT candidates does vary between centers and that there is still a lot of uncertainty on its therapeutic value in this population. All centers agreed that obesity in KT recipients presents a modifiable risk factor with potential treatment options. Despite the increasing number of case series reporting promising results, there is still a lack of high-quality prospective data to prove the efficacy and safety of BS in KT candidates [15]. Furthermore, the effects of BS on graft function and immunosuppressive medication have not yet been deeply investigated [16]. Hence, the first step to reach for a national policy is the interest of most centers to participate in a nationwide prospective study on the topic. The future goal should be that no patient is excluded from KT based solely on an obesity-related criterion without assessment for BS or a multidisciplinary approach.

Certainly, our study has limitations. First, the survey consisted solely of 16 core questions, and we did not perform a second questionnaire round to explore further details. Second, albeit 100% of all centers responded at least once to the query, some centers did not report both the nephrologists’ and surgeons’ perspectives on the questionnaire due to differing house politics. Furthermore, the survey did not distinguish between large and small centers. Therefore, no statements on selection or infrastructure biases can be made. This aspect should be considered in further studies. Last, the study was conducted in Germany, where the allocation system and donor pools differ from those in other countries. Therefore, some statements may not represent the situation for each country due to different policies in transplantation medicine. Additionally, there is no national registry for obese ESRD patients. Therefore, the exact number of patients who are refused KT due to obesity is still unknown. Furthermore, for a long time, access to BS was only granted after strict selection and exhaustion of all conservative measures in Germany. It was not until 2018 that the cumbersome and time-consuming approval process for BS was reformed for patients with particularly severe concomitant and secondary diseases. Therefore, BS was rarely considered for KT candidates before 2018 in Germany.

## Conclusion

Obesity represents a pertinent topic in German KT waiting list policy and BMI limits are still used for KT access. No national policy consensus is currently available and the experience with BS in the field of KT is relatively low with only few centers considering BS for obesity treatment by default. However, there is an emerging trend on the horizon. Most heads of German transplant centers agree that BMI thresholds are improper selection criteria for KT, and BS may play a relevant role in a multimodal obesity treatment in KT candidates. Therefore, a national multicenter study seems to be the next proper step to address the concerns regarding the risks and safety of BS in KT candidates and could help to frame a unified national policy on this topic.

## Supplementary Information

Below is the link to the electronic supplementary material.

Supplementary file1 (DOCX 43 KB)

## Abbreviations

BS: Bariatric surgery
CKD: Chronic kidney disease
DGF: Delayed graft functions
ESRD: End-stage renal disease
KT: Kidney transplantation
PNF: Primary non-function
