# Supplementary material for: Body Mass Index Thresholds and the Use of Bariatric Surgery in the Field of Kidney Transplantation in Germany
Source: Obes Surg. 2022 Mar 19;32(5):1641–8. doi: 10.1007/s11695-022-06000-4 (PMC8986752; doi:10.1007/s11695-022-06000-4)
Supplement: Supplementary file 1 — Supplementary file1 (DOCX 43 KB) [file 11695_2022_6000_MOESM1_ESM.docx]

**Supplementary File 1 – Questionnaire with all responses.**

**1) Does obesity in ESRD patients present a relevant issue in your center? (Figure 2;A)**

|  | Individual  (n = 51) | Surgery  (n = 28) | Nephrology  (n = 23) |
| --- | --- | --- | --- |
| Yes | 49 (96.1) | 27 (96.4) | 22 (95.7) |
| No | 2 (3.9) | 1 (3.6) | 1 (4.3) |

P = 1

**2. Does your center have a policy or standard operating procedure for ESRD patients with obesity regarding inclusion on the kidney transplant waiting list? (Figure 2;B)**

|  | Individual  (n = 51) | Surgery  (n = 28) | Nephrology  (n = 23) |
| --- | --- | --- | --- |
| Yes | 34 (66.7) | 18 (64.3) | 16 (69.6) |
| No | 17 (33.3) | 10 (35.7) | 7 (30.4) |

P = 0.691

**3. What is the upper limit BMI at your center for considering patients with ESRD for kidney transplantation evaluation? (Figure 2;C)**

|  | Individual  (n = 51) | Surgery  (n = 28) | Nephrology  (n = 23) |
| --- | --- | --- | --- |
| 30 | 1 (2) | 0 (0) | 1 (4.3) |
| 32 | 4 (7.8) | 2 (7.1) | 2 (8.7) |
| 35 | 14 (27.5) | 6 (21.4) | 8 (34.8) |
| 40 | 3 (5.9) | 3 (10.7) | 0 (0) |
| None | 28 (54.9) | 16 (57.1) | 12 (52.2) |
| N/A | 1 (2) | 1 (3.6) | 0 (0) |

P = 0.367

**4. What BMI is the upper sole exclusion criterion for patients with ESRD to be eligible for kidney transplantation (status "transplantable") at your center? (Figure 2;D)**

|  | Individual  (n = 51) | Surgery  (n = 28) | Nephrology  (n = 23) |
| --- | --- | --- | --- |
| 30 | 4 (7.8) | 1 (3.6) | 3 (13.0) |
| 35 | 23 (45.1) | 12 (42.9) | 11 (47.8) |
| 40 | 8 (15.7) | 5 (17.9) | 3 (13.0) |
| Individual | 4 (7.8) | 3 (10.7) | 1 (4.3) |
| None | 12 (23.5) | 7 (25.0) | 5 (21.7) |

P = 0.661

**5. Does the BMI present an appropriate criterion for the selection of potential kidney transplant candidates? (Figure 2;E)**

|  | Individual  (n = 51) | Surgery  (n = 28) | Nephrology  (n = 23) |
| --- | --- | --- | --- |
| Yes | 29 (56.9) | 16 (57.1) | 13 (56.5) |
| No | 22 (43.1) | 12 (42.9) | 10 (43.5) |

P = 0.964

**6. if not, which of the following parameters do you consider as better criteria? (Multiple answers possible)(Figure 2;F)**

|  | Individual  (n = 22) | Surgery  (n = 12) | Nephrology  (n = 10) |
| --- | --- | --- | --- |
| Waist-to-hip ratio | 14 (63.6) | 9 (75) | 5 (50) |
| Waist circumference | 19 (86.4) | 11 (91.7) | 8 (80) |
| Body fat percentage | 9 (40.9) | 6 (50) | 3 (30) |
| Individual | 1 (4.5) | 0 (0) | 1 (10) |
| Other | 2 (9.1) | 1 (8.3) | 1 (10) |

**7.** **Are weight loss programs useful measures for patients with obesity before kidney transplantation? (Figure 3;A)**

|  | Individual  (n = 51) | Surgery  (n = 28) | Nephrology  (n = 23) |
| --- | --- | --- | --- |
| Unclear | 2 (3.9) | 0 (0) | 2 (8.7) |
| Yes | 48 (94.1) | 28 (100) | 20 (87.0) |
| No | 1 (2) | 0 (0) | 1 (4.3) |

P = 0.144

**8.** **Which weight reduction measures do you consider appropriate in ESRD patients to treat obesity prior to kidney transplantation? (Multiple answers possible) (Figure 3;B)**

|  | Individual  (n = 51) | Surgery  (n = 28) | Nephrology  (n = 23) |
| --- | --- | --- | --- |
| Activity programs | 48 (94.1) | 27 (96.4) | 21 (91.3) |
| Nutritional counseling | 49 (96.1) | 28 (100) | 21 (91.3) |
| Bariatric surgery | 47 (92.2) | 25 (89.3) | 22 (95.7) |

**9.** **Which weight loss measures does your center offer to ESRD patients with obesity in preparation for kidney transplantation? (Multiple answers possible) (Figure 3;C)**

|  | Individual  (n = 51) | Surgery  (n = 28) | Nephrology  (n = 23) |
| --- | --- | --- | --- |
| Activity programs | 34 (66.7) | 21 (75) | 13 (56.5) |
| Nutritional counseling | 45 (88.2) | 28 (100) | 17 (73.9) |
| Bariatric surgery | 37 (72.5) | 21 (75) | 16 (69.6) |

**10.** **Do you treat all waiting list patients, who are above the BMI threshold with bariatric surgery at your center?**  **(Figure 3;D)**

|  | Individual  (n = 51) | Surgery  (n = 28) | Nephrology  (n = 23) |
| --- | --- | --- | --- |
| Yes | 21 (41.2) | 12 (42.9) | 9 (39.1) |
| No | 29 (56.9) | 15 (53.6) | 14 (60.9) |
| N/A | 1 (2) | 1 (3.6) | 0 (0) |

P = 0.612

**11.** **Which bariatric surgery is the most suitable in the context of kidney transplantation? (Figure 3;E)**

|  | Individual  (n = 48) | Surgery  (n = 28) | Nephrology  (n = 23) |
| --- | --- | --- | --- |
| Roux-Y-gastric bypass | 3 (6.3) | 2 (8) | 1 (4.3) |
| Sleeve gastrectomy | 37 (77.1) | 19 (76) | 18 (78.3) |
| Both | 3 (6.3) | 3 (12) | 0 (0) |
| N/A | 5 (10.4) | 1 (4) | 4 (17.4) |

P = 0.166

**12. Which bariatric surgery strategy should be preferred in ESRD patients with obesity? (Figure 3;F)**

|  | Individual  (n = 51) | Surgery  (n = 28) | Nephrology  (n = 23) |
| --- | --- | --- | --- |
| Bariatric surgery **before** kidney transplantation | 45 (88.2) | 22 (78.6) | 23 (100) |
| Bariatric surgery **after** kidney transplantation | 1 (2) | 1 (3.6) | 0 (0) |
| No bariatric surgery | 2 (3.9) | 2 (7.1) | 0 (0) |
| Other | 3 (5.9) | 3 (10.7) | 0 (0) |

P = 0.134

**13. What may be the benefits of bariatric surgery before kidney transplant? (Multiple answers possible) (Figure 4;A)**

|  | Individual  (n = 51) | Surgery  (n = 28) | Nephrology  (n = 23) |
| --- | --- | --- | --- |
| Weight loss | 42 (82.4) | 24 (85.7) | 18 (78.3) |
| Reduction in immunological complications | 10 (19.6) | 7 (25) | 3 (13) |
| Reduction in infections | 28 (54.9) | 17 (60.7) | 11 (47.8) |
| Reduction of surgical complications | 43 (84.3) | 21 (75) | 22 (95.7) |
| Lower delayed graft function incidence | 16 (31.4) | 10 (35.7) | 6 (26.1) |
| Other | 3 (5.9) | 1 (3.6) | 2 (8.7) |
| No advantages | 2 (3.9) | 2 (7.1) | 0 (0) |

**14. What concerns do you have about bariatric surgery in the context of kidney transplantation? (Multiple answers possible) (Figure 4;B)**

|  | Individual  (n = 51) | Surgery  (n = 28) | Nephrology  (n = 23) |
| --- | --- | --- | --- |
| Malnutrition | 7 (13.7) | 2 (7.1) | 5 (21.7) |
| Increases morbidity on dialysis | 9 (17.6) | 5 (17.9) | 4 (17.4) |
| Affects pharmacokinetics of immunosuppressive therapy | 20 (39.2) | 10 (35.7) | 10 (43.5) |
| Increases morbidity after kidney transplantation | 5 (9.8) | 3 (10.7) | 2 (8.7) |
| Increases perioperative complications after kidney transplantation | 4 (7.8) | 1 (3.6) | 3 (13) |
| Increased perioperative complications of bariatric surgery | 4 (7.8) | 2 (7.1) | 2 (8.7) |
| No survival benefit | 3 (5.9) | 1 (3.6) | 2 (8.7) |
| None | 17 (33.3) | 12 (42.9) | 5 (21.7) |
| Other | 2 (3.9) | 0 (0) | 2 (8.7) |

**15. How many patients underwent kidney transplantation after bariatric surgery at your center? (Figure 4;C)**

|  | Individual  (n = 51) | Surgery  (n = 28) | Nephrology  (n = 23) |
| --- | --- | --- | --- |
| <5 | 29 (56.9) | 14 (50) | 15 (65.2) |
| 5-10 | 6 (11.8) | 3 (10.7) | 3 (13) |
| 11-24 | 0 (0) | 0 (0) | 0 (0) |
| >25 | 5 (9.8) | 3 (10.7) | 2 (8.7) |
| None | 11 (21.6) | 8 (28.6) | 3 (13) |

P = 0.565

**16. Are you interested in participating in a prospective multicenter study on obesity in the context of kidney transplantation? (Figure 4;D)**

|  | Individual  (n = 51) | Surgery  (n = 28) | Nephrology  (n = 23) |
| --- | --- | --- | --- |
| Yes | 46 (90.2) | 27 (96.4) | 19 (82.6) |
| No | 5 (9.8) | 1 (3.6) | 4 (17.4) |

P = 0.162
